# Regulated hAAT Expression from a Novel rAAV Vector and Its Application in the Prevention of Type 1 Diabetes

**DOI:** 10.3390/jcm8091321

**Published:** 2019-08-28

**Authors:** Hongxia Ma, Yuanqing Lu, Keith Lowe, Lonneke van der Meijden-Erkelens, Clive Wasserfall, Mark A. Atkinson, Sihong Song

**Affiliations:** 1 College of Animal Science and Technology, Jilin Agricultural University, Changchun 130118, China; 2 Department of Pharmaceutics, University of Florida, Gainesville, FL 32610, USA; 3 Department of Pathology, Immunology and Laboratory Medicine, Diabetes Institute, College of Medicine, University of Florida, Gainesville, FL 32610, USA

**Keywords:** serine proteinase inhibitor (SERPIN), alpha 1 antitrypsin (AAT), autoimmune disease, type 1 diabetes (T1D), gene therapy, recombinant adeno-associated virus (rAAV), tet-on promoter

## Abstract

We, and others, have previously achieved high and sustained levels of transgene expression from viral vectors, such as recombinant adeno-associated virus (rAAV). However, regulatable transgene expression may be preferred in gene therapy for diseases, such as type 1 diabetes (T1D) and rheumatoid arthritis (RA), in which the timing and dosing of the therapeutic gene product play critical roles. In the present study, we generated a positive feedback regulation system for human alpha 1 antitrypsin (hAAT) expression in the rAAV vector. We performed quantitative kinetics studies in vitro and in vivo demonstrating that this vector system can mediate high levels of inducible transgene expression. Transgene induction could be tailored to occur rapidly or gradually, depending on the dose of the inducing drug, doxycycline (Dox). Conversely, after withdrawal of Dox, the silencing of transgene expression occurred slowly over the course of several weeks. Importantly, rAAV delivery of inducible hAAT significantly prevented T1D development in non-obese diabetic (NOD) mice. These results indicate that this Dox-inducible vector system may facilitate the fine-tuning of transgene expression, particularly for hAAT treatment of human autoimmune diseases, including T1D.

## 1. Introduction

Type 1 diabetes (T1D) is an autoimmune disease that results in the destruction of insulin-producing islet cells in the endocrine pancreas [1]. Despite the introduction of modified insulin analogues, along with advances in glucose monitoring and insulin delivery technology, the majority of patients with T1D fail to achieve target glycemic control. To date, there is no cure for T1D, and development of an effective and safe therapy is urgently needed. Many lines of evidence indicate that antigen-presenting cells (APC), particularly dendritic cells (DC), are pathologically active in orchestrating the process of insulitis [2]. APCs within islets respond to micro-environmental stimuli and subsequently activate β-cell-reactive T cells, which then infiltrate and destroy islet cells, resulting in T1D. The cellular infiltrate is heterogeneous in composition, with a predominance of T cells along with varying percentages of macrophages, DCs, natural killer (NK) cells, and B lymphocytes. Substantial evidence demonstrates that both CD4^+^ T helper and CD8^+^ T cytotoxic lymphocytes play a role in the development of the disorder [3,4,5,6,7,8]. Recent studies have shown that increases of CD4^+^CD25^+^ regulatory T cells (Tregs) can prevent autoimmune diabetes in the non-obese diabetic (NOD) mouse model of T1D [9,10,11,12]. It has been shown that both direct cytotoxic and indirect cytokine-dependent mechanisms are responsible for β-cell apoptosis [13]. Granzymes (extrinsic from CD8+ T cells) and caspases (intrinsic from the beta cell, i.e., caspase-3) are key players in controlling the events leading to cell apoptosis, and their inhibition by proteinase inhibitors can protect against islet cell apoptosis [14]. Although multiple factors are thought to contribute to T1D, an imbalance of the immunoregulatory pathways appears to play an important role in disease development. Therefore, immune regulatory approaches hold great potential for the prevention of this disease. Gene therapy with the anti-inflammatory cytokine interleukin-10 (IL-10) induces Tregs and prevents T1D in NOD mice [15,16], setting the stage for the development of other immunoregulatory therapies to prevent or reverse autoimmunity.

Human alpha 1 antitrypsin (hAAT) is a multifunctional protein with anti-proteinase, anti-inflammatory, and immunoregulatory properties [17]. As a member of the serine protease inhibitor (SERPIN) family, a well-known function of hAAT is the inhibition of serine proteinases, including neutrophil elastase, proteinase 3, cathepsin G, thrombin, trypsin, and chymotrypsin [18]. Studies have shown that hAAT inhibits caspase-1 and caspase-3 and, as a result, prevents apoptosis [14,19,20]. Additionally, hAAT directly interacts with the cell surface receptors, TNFR1 and TNFR2, and thereby regulates target cell gene expression [21]. Increasing evidence indicates that hAAT plays an important role in many biological processes, including modulating the immune system. We found that *hAAT (SERPINA1)* gene therapy prevented T1D in the NOD mouse model [22]. Subsequent studies have further demonstrated that hAAT therapy has a therapeutic effect in several disease models, including T1D [14,22,23,24], islet cell transplantation [25,26], rheumatoid arthritis (RA) [27,28], graft versus host disease (GVHD) [29], stroke [30], bone loss [31], lupus [32], and aging models [33]. Remarkably, in all autoimmune disease models, hAAT treatment significantly prevented tissue damage (reducing insulitis in NOD mice, joint destruction in the collagen-induced arthritis (CIA) mouse model, and nephritis in the Murphy Roths Large lymphoproliferative (MRL/lpr) mouse model of lupus) and reduced autoantibody levels, including insulin autoantibodies (IAA) in NOD mice, anti-collagen antibody in CIA mice, and antinuclear antibody (ANA) and anti-dsDNA antibodies in MRL/plr mice. Our recent studies showed that hAAT treatment inhibits DC maturation and function, including reduced production of type 1 interferon (IFN-I) and other proinflammatory cytokines [32].

Adeno-associated virus (AAV) is a single-stranded DNA parvovirus with a 4.7 kb genome and three capsid proteins to form a 20 nm particle. Recombinant AAV (rAAV) has several unique features making it an excellent vector for therapeutic gene delivery [34]; it is nonpathogenic, has low immunogenicity, and carries only three capsid proteins, which induce relatively low responses in the transduced cells when compared to other viruses. The vector does not carry any viral genes; rather, the only viral DNA sequences in the rAAV vector are two inverted terminal repeats (ITRs). In addition, the majority of rAAV DNA remains in episomal forms in non-dividing or long-lived cells (e.g., liver or muscle cells), mediating long-term gene expression. It has been shown that rAAV vectors can provide long-term transgene expression in a wide variety of tissues, including muscle [35,36,37,38,39], lung [40], liver [41,42,43,44], brain [45], and eye [46]. This feature is critical for chronic disease, such as T1D. We previously developed rAAV vectors expressing hAAT under the control of a constitutive promoter [35,43]. Although these vectors have been demonstrated to prevent or ameliorate autoimmunity in several disease models [23,28,31,47], the ability to more precisely control timing and levels of hAAT expression may be critical at different disease stages in order to maximize safety and efficacy for eventual translation to human studies. In order to achieve regulatable *hAAT* gene expression, we developed an inducible rAAV vector using a tetracycline-controlled (tet-on) system. In this study, we characterized the kinetics of *hAAT* expression in response to the regulatory drug doxycycline (Dox), and evaluated the therapeutic efficacy of hAAT induction in pre-diabetic NOD mice.

## 2. Materials and Methods

### 2.1. Animals

C57BL/6 mice and female NOD (NOD/MrkTac) mice were purchased at 4 and 8 weeks of age from Taconic Farms (Germantown, NY, USA). All mice were housed in specific pathogen-free facilities at the University of Florida (Gainesville, FL, USA). The Institutional Animal Care and Use Committee at the University of Florida approved all animal studies. NOD mice were divided into five groups. Group 1: 4 week old mice (*n* = 11) were injected with rAAV1-tet-on-hAAT (3.5 × 10^11^ vg/mouse) and fed with Dox-containing chow (200 mg/kg). Group 2: 8 week old mice (*n* = 6) were injected with rAAV1-tet-on-hAAT (3.5 × 10^11^ vg/mouse) and fed with Dox-containing chow (200 mg/kg). Group 3: 4 week old mice (*n* = 10) were injected with rAAV1-tet-on-hAAT (3.5 × 10^11^ vg/mouse) and fed with chow without Dox. Group 4: 4 week old mice (*n* = 11) were injected with PBS and fed with Dox-containing chow. Group 5: 4 week old mice (*n* = 10) were injected with PBS and fed with chow without Dox. Beginning at 10 weeks of age, mice were monitored weekly for hyperglycemia until they became diabetic, defined as two consecutive (>24 h apart) non-fasting blood glucose levels >240 mg/dL.

### 2.2. Vector Construction, Production, and Administration

In order to generate the rAAV-tet-on-hAAT vector, we used the rAAV-CB-AAT vector as a parental vector. The cytomegalovirus (CMV) enhancer and chicken beta actin promoter in plasmid CB-AAT was removed by Bgl*II* and Eco*RI* digestion and replaced by *the reversed tetracycline transactivator (rtTA)* gene from a plasmid p43rtTA. The resulting intermediate clone contained *rtTA* and *hAAT* genes in a head-to-head manner. The tetracycline responsive elements (seven repeats), flanked by two mini CMV promoters from a commercial plasmid pBI-GL (Clontech, Palo Alto, CA, USA), were inserted between *rtTA* and *hAAT* genes in the intermediate plasmid. In the final vector, rAAV-tet-on-hAAT, *rtTA*, and *hAAT* genes were controlled by a bi-directional promoter, which was doxycycline-inducible and self-accelerated.

### 2.3. Detection of Transgene Expression

Serum hAAT and anti-hAAT were detected by enzyme-linked immunosorbent assay (ELISA), as previously described [48]. For each injection area, we harvested three pieces of muscle and performed immunostaining to detect *hAAT* transgene expression in injected muscle, as previously described [49].

## 3. Results

### 3.1. Construction and In Vitro Evaluation of rAAV1-tet-on-hAAT Vector

**Figure 1:**
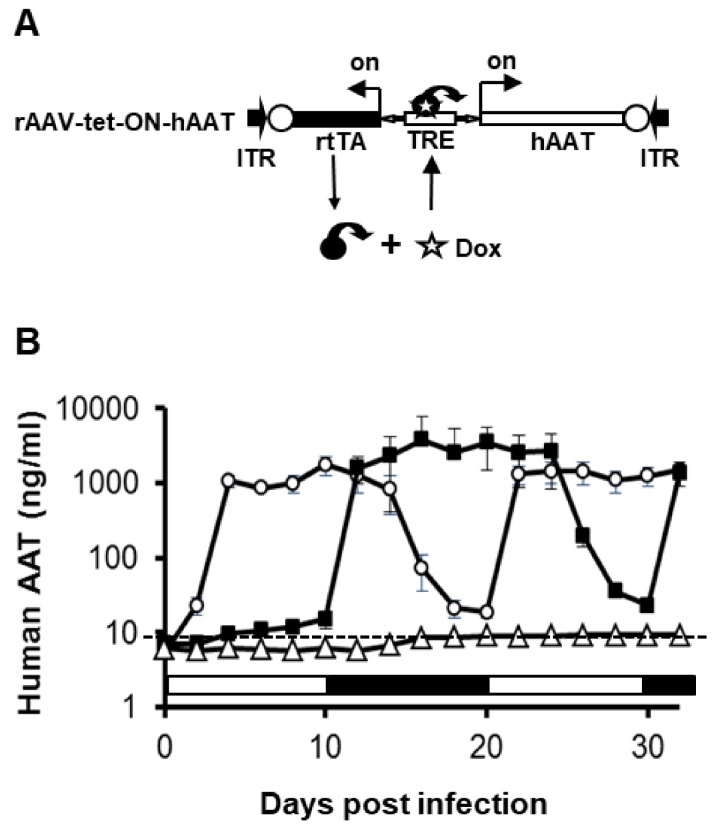
Transgene expression from recombinant adeno-associated virus (rAAV)1-tet-on-hAAT in differentiated C2C12 cells. (**A**) The vector construct. ITR, AAV inverted terminal repeat sequences. TRE, tetracycline response elements. hAAT, *hAAT* cDNA. rtTA, *reversed tetracycline transactivator* gene. (**B**) hAAT levels detected in culture medium. Cell culture medium was collected and replaced every 2 days. The open circle group was cultured in medium with Dox (1 μg/mL) for 10 days (day 0 to day 10 indicated by an open box), and then without Dox for 10 days (day 10 to day 20 indicated by a black box). The DOX treatment cycle was repeated after day 20. The filled square group was cultured in medium without Dox for 10 days (day 0 to day 10 indicated by open box), and then with Dox for 10 days (day 10 to day 20 indicated by black box). This DOX treatment cycle was also repeated. The experiment ended on day 32.

In order to achieve regulated hAAT expression from rAAV vectors, we employed the tet-on systems. Using a bidirectional tet-responsive promoter, which controls both *rtTA* and *hAAT* genes, we generated a regulated hAAT expression vector system with positive feedback (Figure 1A). After construction, we packaged this vector into rAAV1-tet-on-hAAT. In vitro evaluation of regulated transgene expression was performed in differentiated C2C12 cells (mouse myoblasts). As shown in Figure 1B, the addition of Dox (1 µg/mL) resulted in high levels of sustained hAAT expression (1–4 µg/mL). Withdrawal of Dox led to a 50- to 100-fold reduction of hAAT levels in the culture medium. We noted that delayed application of Dox (10 days after infection) resulted in quicker induction of hAAT expression, while withdrawing Dox resulted in comparatively slower reduction of hAAT expression. We also observed leaky expression (~1% of full expression level) in the absence of Dox.

### 3.2. Regulated hAAT Expression from rAAV1-tet-on-hAAT in Muscle

**Figure 2:**
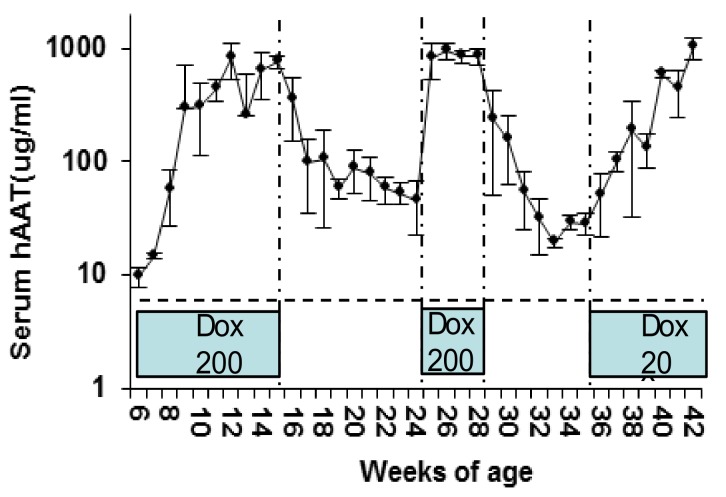
Regulated hAAT levels in C57BL/6 mice. Animals (*n* = 4) received intramuscular (IM) injection of rAAV1-tet-on-hAAT vector (2 × 10^11^ vg/mouse) at 6 weeks of age. Dox-containing chow (200 µg/kg) was provided from 6–15 weeks of age, and replaced by normal food without Dox between 16 and 23 weeks of age. From 24 to 28 weeks of age, animals were fed Dox-containing chow (200 µg/kg). Animals received low Dox-containing chow (20 µg/kg) from 35 to 42 weeks of age, as indicated in the blue boxes.

In order to evaluate the kinetics of the transgene expression in vivo, rAAV1-tet-on-hAAT vector (5 × 10^11^ vg/mouse) was intramuscularly injected into C57BL/6 mice. Mice received Dox-containing food (200 µg/kg food). hAAT serum levels increased gradually and reached steady state 4 weeks after vector injection (Figure 2). The level of serum hAAT (1000 µg/mL) from rAAV1-tet-on-hAAT vector was comparable to that from rAAV1-CB-hAAT vector in previous studies [49]. After the withdrawal of Dox, hAAT levels gradually decreased 50-fold and reached a steady state approximately 7 weeks later (Figure 2). Interestingly, when dosing with Dox resumed, hAAT levels quickly reached and sustained the peak level, indicating the system using a bidirectional promoter can serve as a quick turn-on switch, but after the subsequent withdrawal of Dox, hAAT gradually decreased 50-fold again (Figure 2). The slow turn-off process is likely dependent on the depletion of Dox, the half-life of the mRNA, and hAAT protein in the animal. The remaining hAAT expression 5–6 weeks after withdrawing Dox was 2–5% of full expression and may represent the leaky promoter activity. To test the dosing effect of Dox on the kinetics of transgene expression, low Dox-containing food (20 µg/kg) was used. Intriguingly, low-dose Dox and high-dose Dox chow induced hAAT expression to comparable peak levels, though with delayed kinetics in the low-dose Dox group (Figure 2). Specifically, low-dose Dox induced a gradual increase in serum hAAT over the course of 7 weeks. These data indicate that a Dox dosing regimen can be used to control the kinetics of hAAT levels in mice.

### 3.3. Prevention of Type 1 Diabetes in NOD Mice by rAAV1-tet-on-hAAT and Dox

**Figure 3:**
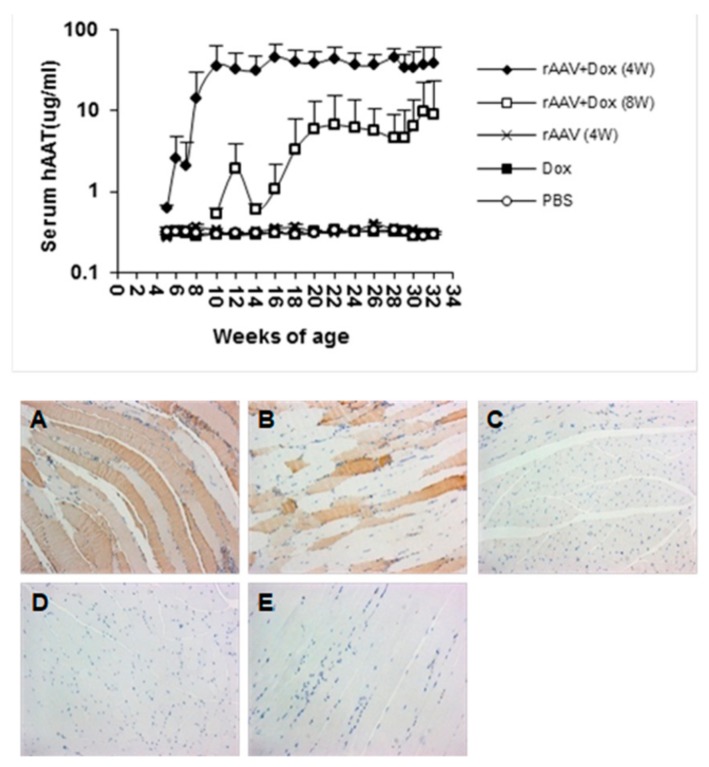
Transgene (*hAAT*) expressed from rAAV1-tet-on-hAAV in non-obese diabetic (NOD) mice. Top panel: serum hAAT levels detected by ELISA. Bottom panel: representative images of immunostaining for hAAT. Muscle sections from different groups: (**A**) rAAV + Dox (4 W) mice received rAAV vector and Dox-containing food (*n* = 11); (**B**) rAAV + Dox (8 W) mice received rAAV and Dox (*n* = 6); (**C**) rAAV (4 W) mice received rAAV only, without Dox (*n* = 10); (**D**) Dox mice received Dox alone without rAAV vector (*n* = 11); (**E**) Mice received phosphate-buffered saline (PBS) injections (*n* = 10).

**Figure 4:**
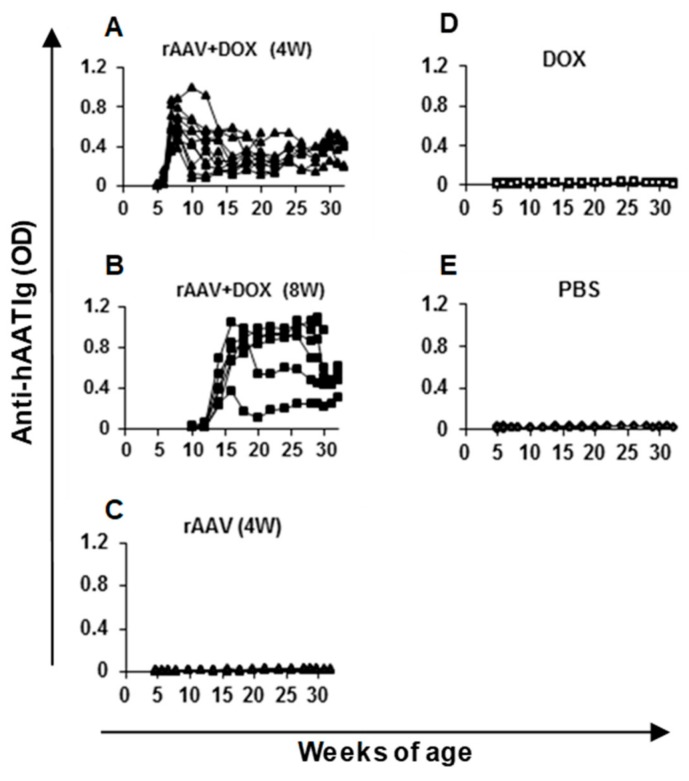
Anti-hAAT IgG levels in NOD mice. Serum samples from each mouse were diluted 100 times and subjected to ELISA. Each line represents data (optical density, OD) from an individual animal. The group labels are the same as indicated in Figure 3.

**Figure 5:**
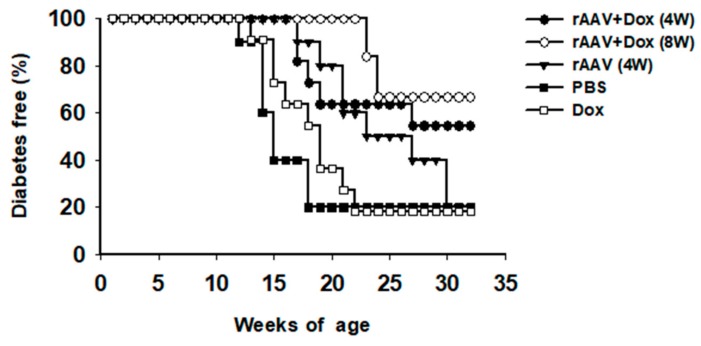
Life table analysis for diabetes development. All animals were monitored weekly for hyperglycemia. If two consecutive (>24 h apart) non-fasting blood glucose levels (>240 mg/dL) were recorded, the animal was defined as diabetic and was removed from the group. * *p* < 0.05, rAAV + Dox (4 W) or rAAV + Dox (8 W) vs. PBS or Dox.

In order to test this vector system for the prevention of T1D, cohorts of NOD mice were intramuscularly injected with rAAV1-tet-on-hAAT vector at 4 or 8 weeks of age. After vector injection, mice received Dox-containing chow (200 µg/kg food). Control groups received saline injection, Dox alone, or rAAV1-tet-on-hAAT injection alone (without Dox). Treatment with rAAV-tet-on-hAAT plus Dox at 4 weeks resulted in relatively higher transgene expression (Figure 3) and lower anti-hAAT antibody responses (Figure 4) than the same treatment initiated at 8 weeks. Importantly, both treatments resulted in a significant prevention of T1D development compared to control groups (Figure 5).

## 4. Discussion

Transgene expression levels from gene therapy vectors are critical for the efficacy and safety of the treatment, particularly compared to intermittent injection of the therapeutic protein that results in variable drug levels in circulation. High and sustained levels of transgene expressions have been achieved in many gene therapy studies [35,43]. In some disease settings, consecutive promoters have been used resulting in sustained transgene expression, which is preferred for the treatment of some genetic diseases. However, in many cases, transgene expression from gene therapy vectors may need to be controlled or regulated. For example, the need for therapeutic protein (i.e., the transgene product) may be different at different stages of inflammatory or autoimmune disease, or an off mechanism may be desirable for safety reasons. Several strategies for controlling transgene expression have been developed, including tet-on/off systems. However, quantitative analysis of the kinetics of transgene expression remain to be investigated. Manfredsson et al. characterized an rAAV-based bicistronic tet-off system in the brain, and showed that transgene expression was highly sensitive to dietary Dox [50]. We developed a single rAAV vector, carrying both regulatory (*rtTA*) and therapeutic (*hAAT*) genes, using a bidirectional promoter to achieve Dox-regulated hAAT expression, and showed its therapeutic potential for the treatment of RA [27]. In the current study, we quantitatively characterized the positive feedback rAAV vector system using Dox to control hAAT expression, and showed the protective effect against spontaneous autoimmune diabetes in NOD mice. Although the level of hAAT from our rAAV vector can be changed by Dox, it is relatively stable compared to fluctuating levels with hAAT protein administration; therefore, it should be considered an effective drug delivery system. In this study, we observed leaky hAAT expression in the absence of Dox (1–5%). Since hAAT is a major component of human serum (normal range = 1–2 mg/mL) and can be safely upregulated 3–4-fold under inflammatory conditions, we do not anticipate this low level of leaky expression to be a concern in the clinical setting [18,51]. There are some limitations to the present study. For example, the sample sizes were relatively small in some of the groups, and therapeutic effects observed in the NOD mouse model often do not translate directly to human diabetes. Nonetheless, our results indicate that this vector system may be useful to fine-tune hAAT levels for a safe and efficient *hAAT* gene therapy to treat autoimmune and chronic inflammatory diseases.

Our results also showed that hAAT expression levels appeared to be inversely correlated with anti-hAAT antibody levels in NOD mice. Gene delivery at 4 weeks of age resulted in high levels of hAAT and lower anti-hAAT levels. In contrast, gene delivery at 8 weeks of age resulted in lower peak levels of hAAT, but high and persistent levels of anti-hAAT antibody. This could possibly be related to the fact that NOD mice develop autoimmunity as they mature, and are prone to developing antibody-mediated immunity against human proteins administered in experimental settings [52]. Consistent with our previous observations [35], these results clearly demonstrate an effect of host immune responses against the transgene product. It is notable that this effect is commonly seen in various autoimmune disease models, wherein relatively lower levels of hAAT and higher levels of anti-hAAT can be detected than in C57BL/6 mice. It is well known that the immune response against transgene products is AAV vector serotype dependent [48]. We have shown that rAAV1 vectors infect dendritic cells (DCs) effectively and induce a strong immune response, while rAAV8 vectors fail to infect DCs and induce immune tolerance to the transgene products in several disease models [28,31,47,48]. Future studies using different rAAV serotypes of this vector expressing Dox-regulated hAAT will provide more detailed insight related to the immune response against regulated hAAT expression. It should be noted that administration of hAAT to AAT-deficient patients does not induce an antibody response to hAAT [53]. Therefore, the immune response to hAAT observed here should not be a concern in clinical settings. Importantly, the therapeutic effect of hAAT in the prevention of T1D can be observed with lower hAAT levels from rAAV vectors administered at later time points. It is possible that the sustained kinetics of hAAT expression contribute to the therapeutic effect. In addition, Dox as an antibiotic affecting intestinal flora may contribute to the prevention of T1D [54,55]. Although Dox treatment alone did not significantly prevent T1D in this study, it is possible that Dox and hAAT have a combined effect. In this case, Dox has dual roles as a therapeutic drug and an inducer for *hAAT* gene expression [27]. Therefore, this vector system provides a combination therapy. Future studies optimizing the dose of rAAV vector and Dox may further improve this potential synergistic therapeutic effect.

## 5. Conclusions

In summary, we have demonstrated several unique features of this vector system: (1) The positive feedback regulation system can result in high levels (~1000 µg/mL) of transgene expression, which is comparable to the levels achieved under control of the CMV enhancer and chicken β-actin (CB) promoter; (2) transgene expression can be turned on quickly by high doses of Dox and turned on slowly by low doses of Dox, providing a controllable on-switch, which will be important for translation to human studies; (3) withdrawal of Dox resulted in a slow decline followed by sustained low levels of transgene expression. These tunable kinetics allow for the long-term control of medium or low expression levels by adjusting the Dox dose regimen to mitigate the risk of negative side effects. Although leaky expression is unfavorable in some gene therapy, low levels of long-term hAAT expression may be beneficial for the treatment of chronic diseases, such as T1D or RA.
